# Wernicke Syndrome: Case Report and Literature Review of Contributing Factors—Can Malpractice Dynamics Be Identified?

**DOI:** 10.3390/jcm13030716

**Published:** 2024-01-26

**Authors:** Donatella Mangione, Alessandra Vassiliadis, Giuseppe Gullo, Cetty Gullo, Gaspare Cucinella, Renato Venezia, Simona Zaami

**Affiliations:** 1 Department of Obstetrics and Gynecology, A.O.U.P. P. Giaccone, University of Palermo, 90127 Palermo, Italy; donatella.mangione@unipa.it (D.M.); alessandra.vassiliadis@unipa.it (A.V.); cettygullo1994@gmail.com (C.G.); renato.venezia@unipa.it (R.V.); 2 Department of Obstetrics and Gynecology, Villa Sofia Cervello Hospital, University of Palermo, 90146 Palermo, Italy; gullogiuseppe@libero.it (G.G.); gaspare.cucinella@unipa.it (G.C.); 3 Department of Anatomical, Histological, Forensic and Orthopedic Sciences, Sapienza University of Rome, 00198 Rome, Italy

**Keywords:** Wernicke encephalopathy (WE), Hyperemesis Gravidarum (HG), thiamine, ataxia, guidelines, medicolegal standards

## Abstract

Wernicke Encephalopathy (WE) is a neurological acute syndrome related to vitamin B1 deficiency and is relatively common in patients with chronic alcoholism. In the case of Hyperemesis Gravidarum, thiamine body stores become unable to meet the increased demand, resulting in acute deficiency. WE is associated with typical clinical and radiological findings. Treatment pathways rely on thiamine replacement. The case herein reported is centered around a 33-year-old diabetic patient at 12 weeks of gestation, with WE due to hyperemesis gravidarum. The disease manifested itself with weakness, mental confusion, headache, and impaired vision. The diagnosis was established after the detection of typical findings by MRI. Thirty days after therapy was started, most of the patient’s neurological disorders were resolved. The patient was discharged 40 days later with instructions to continue daily thiamine supplementation. The pregnancy outcome was good. Unfortunately, mild ataxia persisted in 2-year follow-up as a long-term consequence. When diagnosed and treated, WE has a favorable prognosis. However, roughly 80% of patients experience memory loss, which may continue for a long time, while gait disorders reportedly affect about 35% of patients. Mild ataxia and dysmetria may persist, too. We reviewed the scientific literature on WE in women with HG until February 2023. Hardly any authors report data on long-term sequelae. Our report emphasizes how important it is to take into consideration this complication in clinical practice, referring to published guidelines and recommendations. Neurological maternal sequelae can demonstrably persist despite early diagnosis and appropriate management. For this reason, a long-term follow-up is recommended. Wernicke syndrome management cannot yet rely on well-established conclusive guidelines; hence, a cautionary approach ought to be prioritized in order to ensure medicolegal soundness.

## 1. Introduction

Wernicke encephalopathy (WE) is an acute neurological disorder resulting from acute or chronic deficiency of vitamin B1 (thiamine). Such a clinical condition is characterized by a triad of ataxia, mental confusion, and ophthalmoplegia and was described for the first time by Carl Wernicke in 1881 as “polioencephalitis hemorrhagica superioris” [1] and subsequently as “polyneuritic psychosis” by Sergej Korsakoff in 1887. Wernicke–Korsakoff Syndrome (WKS) is common among alcohol abusers, whereas WE, not alcohol-related and caused by thiamin depletion only, is rather rare and often misdiagnosed: it can occur in association with malnutrition, prolonged starvation, anorexia, total parenteral nutrition, gastrointestinal disorders, thyrotoxicosis, malignant disorders, chronic kidney diseases, dialysis, and organ transplantation, and it could occur in association with hyperemesis gravidarum [2,3,4]. Such features make non-alcoholic WKS often linked to bariatric surgery, which may result in nutritional malabsorption in obese patients. The clinical diagnosis of thiamine deficiency frequently occurs with no biochemical confirmation due to difficulties associated with the very nature of thiamine compounds, which are, in fact, photosensitive and can only keep their stability for just a few hours at room temperature. All specimens therefore have to be carried at low temperatures and not exposed to light, to then be frozen and stored [5].

The connection with hyperemesis gravidarum was first described in 1939 by Harold Sheehan. Nevertheless, due to the rare nature of such a complication, specific research findings are still somewhat inconclusive [4]. Nausea and vomiting affect about 80% of pregnancies. Hyperemesis gravidarum (HG) occurs in about 3% of pregnancies and is a rather common reason for hospitalization in early pregnancy [6,7,8]. HG complications include dehydration, vitamin deficiencies, and neurological disorders. In most severe cases, liver and kidney failure, immunodepression, and neuropsychiatric involvement till Korsakoff syndrome could occur. An early diagnosis of WE is crucial. The case herein reported is centered around a 33-year-old diabetic patient with WE at 12 weeks of gestational age.

## 2. Materials and Methods

### 2.1. Patient

A 33-year-old patient with a history of RPL (3 miscarriages), inherited thrombophilia (C677T MTHFR without hyperhomocysteinemia), and gestational diabetes (GD) came to our obstetric emergency room at 12 weeks and 3 days of gestational age reporting asthenia, confusion, headache, and decreased vision after repeated vomiting episodes from the previous week. She reported repeated vomiting episodes from the previous week. Obstetrical emergencies were excluded, and fetal status was reassuring at US. 

### 2.2. Clinical Assessment

**Figure 1:**
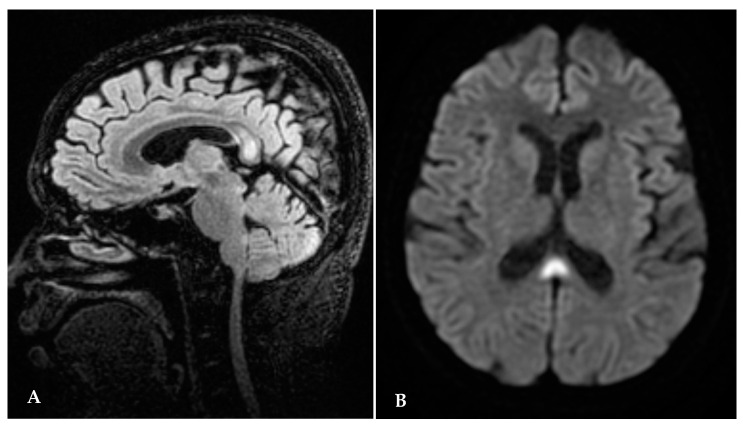
(**A**,**B**) Brain MRI after admission: abnormal signal in the splenium of corpus callosum.

Following admission, the patient was referred first to a diabetologist, who diagnosed “overt diabetes”. This is not a coincidence since thiamine demand increases in metabolism of carbohydrates. Immediately after the diabetologic consultation, patient started insulin therapy and glucose monitoring, after which she underwent a full eye examination, which showed vertical downbeat nystagmus, papilledema, an oval retinal hemorrhage on inferotemporal vascular arcade; all such signs point to a pattern of central nervous system involvement. After the ophthalmological examination, the patient underwent a brain MRI, which showed an abnormal signal in the splenium of corpus callosum and a symmetrical hyperintensity in thalamic and periaqueductal areas (Figure 1A,B), all findings indicative of WE. The patient then underwent a neurological examination, which showed ataxia with impaired walking and nystagmus in all directions of gaze.

### 2.3. Imaging 

Brain MRI performed after the admission showed an abnormal signal in the splenium of corpus callosum and a symmetrical hyperintensity in thalamic and periaqueductal areas (Figure 1). 

**Figure 2:**
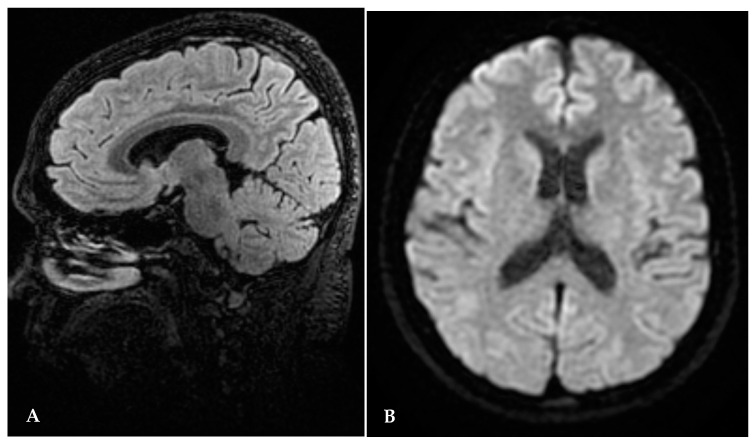
(**A**,**B**) Brain MRI seven months after thiamine start: disappearance of abnormal signal.

A second brain MRI, performed seven months after the beginning of thiamine replacement and one month after delivery, showed hyperintensity to have completely disappeared in the splenium of corpus callosum in thalamic and periaqueductal areas (Figure 2A,B). 

### 2.4. Laboratory 

Laboratory tests showed the following: hyperglycemia (272 mg dL), hypokalemia (2.5 mmol/L), increase in ALT (54 U/L) and GGT (55 U/L), drop in total protein levels (50 mg/dL), presence of glycosuria (70 mg/dL), ketonuria (more than 150 mg/dL), and proteinuria (70 mg/dL). The other blood tests appeared normal, as was the thyroid function.

### 2.5. Treatment 

At first, hyperglycemia and hypokalemia were corrected with insulin administration and KCl i.v. administration, respectively. 

Due to the strong suspicion of WE, thiamine replacement was initiated with a loading dose of 500 mg iv every 8 h for 2 days, then reduced to 250 mg iv a day for 5 days, and finally maintained with 300 mg orally a day.

## 3. Results

Hypokalemia, nausea, and vomiting disappeared after two days of treatment. Almost all other symptoms resolved after one week, but headache and diplopia persisted. After nausea and vomiting, ataxia and mental confusion showed an improvement. An early neurological re-evaluation did not detect any significant changes compared to the first examination, whereas a second ophthalmological exam found a significant regression of retinal hemorrhage. During her hospital stay, fetal US monitoring was performed, which ruled out fetal anomalies. 

After 30 days from the beginning of thiamine replacement therapy, the patient’s clinical status was good, and most of her neurological disorders were resolved. The patient was discharged after 40 days of hospitalization with instructions to continue oral daily thiamine supplementation until delivery and to undergo prenatal follow-up. She was then admitted, at 19 weeks of gestational age, to the “high risk” pregnancy outpatient clinic, where follow-up was performed by gynecologists together with diabetologists. Clinical, obstetrics, and laboratory checks were performed monthly. The patient continued insulin therapy throughout her pregnancy with a good glycemic balance. 

At second-trimester ultrasound (US) screening, no fetal anomalies were detected. After approximately 3 months from WE onset, at neurological follow-up, the patient reported an improvement in her symptoms and total resolution of headache, with persistence of ataxic gait, although she was able to walk with the use of a cane. The ophthalmological conditions improved progressively, too, with complete resolution of papilledema and intraretinal hemorrhage; nystagmus persisted in extreme gaze positions only. Third-trimester fetal ultrasound screening estimated an 1800 g fetal weight at the 80th percentile of growth curves, with a maximum task of amniotic fluid of 8.5 cm in size. The total weight gain was about 20 kg. Neurological examination at term, according to obstetrical indications, did not rule out the feasibility of a vaginal delivery. Furthermore, EEG examination was normal, whereas EMG showed axonal neuropathy to lower limbs. 

### Obstetric Outcome

The patient was admitted to our obstetric department at 38 weeks and 5 days of gestational age, with a diagnosis of premature rupture of membranes (PROM). Labour was inducted by way of vaginal prostaglandins, but a cesarean section was performed because of disengagement of the fetal head: a healthy female was born, with a good Apgar Score (9–9) and a weight of 3370 g, who was entrusted to neonatologists’ care and subsequently discharged after 5 days in good clinical condition. 

The post-partum period was initially characterized by the onset of confabulation and poor collaborative behavior. Laboratory tests were normal, as well as glycemic balance. About four days after delivery, general clinical conditions appeared to have significantly improved, and the patient was oriented and collaborative, albeit some degree of visual disturbances and ataxic gait lingered. Five days after delivery, the gynecological and neurological team decided to discharge the patient, with an indication to continue oral thiamine replacement therapy and to undergo neurological follow-up, which was performed at 3, 6, 12, and 24 months after delivery. Her last neurological examination, representing a 2-year long-term follow-up from the diagnosis of Wernicke Syndrome, unfortunately confirmed the persistence of mild ataxia as probably permanent sequela.

## 4. Discussion

Wernicke encephalopathy is an acute neuropsychiatric disorder characterized by a typical triad: oculomotor disorders (referred to as ophthalmoplegia or nystagmus), cerebellar syndrome (referred to as imbalance or falling), and neuropsychiatric symptoms (referred to as confusion, decreased alertness, altered cognition, or delirium). WE is caused by vitamin B1 deficiency. Prodromal symptoms of severe thiamine deficiency, such as nausea and vomiting, blurred vision, and/or diplopia, generally occur before the onset of WE. Incongruous treatment may result in additional complications, such as Korsakoff Syndrome, anterograde and retrograde amnesia, and confabulation. These complications can lead to persistent disability [9]. 

Thiamine is an essential vitamin. A 25–30 mg body storage of thiamine usually lasts for about 18 days. Vitamin B1 deficiency adversely affects metabolism, especially in brain parenchyma, with its high turnover. Thiamine deficiency leads to a lack of transketolase with consequent pyruvate and lactate brain build-up, resulting in abnormal nerve conduction and cell death via necrosis or apoptosis [10,11]. Cells therefore become unable to produce aerobic energy, and that brings about, as already mentioned, lactic acid and reactive oxygen species buildup. Cell death starts to occur within days. Typical neuroanatomic lesions appear, as a result of such a pathophysiologic process, in the medial thalami and structures of Papez’s circuit, notably the mammillary bodies, tectal plate, periaqueductal area of the midbrain, and periventricular regions of the third ventricle. Atypical manifestations have been reported in the cerebral cortex, cerebellum, and cranial nerve nuclei. The chronic phase of the syndrome resolves into relatively permanent bilateral lesions and enduring global amnesia. The distinctive traits of encephalopathy were described by German neurologist Carl Wernicke 131 years ago, whilst Russian psychiatrist Sergei Korsakoff completed his doctoral dissertation titled “Alcoholic Paralysis,” a few years later, focusing on the circumscribed amnesia reportedly associated with some cases of chronic alcoholism. Over fifty years later, the correlation between such conditions and the specific manifestations thereof was found to be thiamine deficiency [12]. 

The RDA of thiamine is 0.4 mg/1000 kcal, but the need for thiamine in pregnancy grows to 1.5 mg/day [11]. Furthermore, hyperemesis is known to deplete thiamine storage. However, a different genetic susceptibility is reportedly relevant in the pathogenesis, which is likely why WE in pregnancy is rare, unlike hyperemesis. Abnormal liver function tests are present in about 50% of cases of hyperemesis gravidarum, and this association shows an increased risk of WE [13]. 

WE diagnosis is clinically based on the detection of the three telltale signs: oculomotor abnormalities (ophthalmoplegia or nystagmus), cerebellar dysfunction (imbalance or falling), and altered mental status (confusion, decreased alertness, cognition problems, or delirium). 

The classic triad, however, does not arise in all patients. For this reason, Wernicke encephalopathy could be better diagnosed by Caine’s operational criteria. Caine et al. [14] proposed WE diagnosis in the presence of at least two out of four signs: ophthalmoplegia, ataxia, altered mental status or mild memory impairment, and malnourishment. Optic neuropathy is uncommon in vitamin B1 deficiency, but it is usually bilateral and associated with optic disc swelling or papilledema [15,16,17,18]. In a recent review by Reynolds et al. centered on thiamine deficiency in 40 patients, most cases of optic disc swelling (90%; 36/40) were observed in young women (mean age = 28 years). Thiamine deficiency was caused by reduced intake (62.5%; 25/40) due to anorexia or vomiting, and many of these women (60%; 15/25) had hyperemesis gravidarum [18]. The physiopathology of optic neuropathy in thiamine deficiency seems to involve mitochondrial dysfunction [19]. Although WKS has mostly been reported and studied in patients with alcohol use disorders, such a trend appears to be shifting, with a growing body of scientific data accounting for nonalcoholic WKS among medical and surgical patient populations. Cancer may, in fact, cause or amplify thiamine deficiency due to the same set of pathophysiologic mechanisms laid out earlier on, i.e., lower availability, faster usage rates, negatively affected functioning dynamics, and abnormally high losses [20]. Cancer patients have, in fact, been found to be more likely to be thiamine-deficient, and WKS has also been reportedly diagnosed in such patients as well. Two distinct studies have taken into account and assessed the prevalence of WKS in subjects who passed away after bone marrow transplants, finding a broad 6–33% range [21,22]. Alcohol-unrelated Wernicke Syndrome often goes undetected due to the difficulty of performing thiamine tests and the relatively low levels of awareness among clinicians; such an issue entails extra risks since it can delay diagnosis until the condition progresses and manifests itself in one or more of Wernicke’s triad features [23]. In fact, isolated optic disc swelling, particularly linked to milder cases of thiamine deficiency, may go undetected until further development of additional signs of neurological disease. Such a scenario may, in turn, bring about detection and publication biases for more severe cases. Underlying thiamine deficiency has been significantly associated with distinctive manifestations such as T2/FLAIR alterations in the thalamus, periaqueductal grey matter and/or mamillary bodies (revealed by MRI scan), hemorrhagic disc swelling, and normal lumbar puncture opening pressure. Such findings seem to agree with widely acknowledged thiamine deficiency-associated traits detectable by MRI, along with vision loss widely reported. Vitamin supplementation is reportedly highly effective and beneficial in such patients [22,23].

A WE diagnosis needs to be confirmed by MRI, which usually shows a symmetric T1, T2, and T2 flair hyperintensity in the mammillary body, medial thalamus, and periventricular and periaqueductal regions [24]. The neurological features arise from anomalies that develop in specific brain regions. Abnormalities in the thalamomesencephalic areas can cause a disturbance in the level of consciousness, while ophthalmoplegia and ataxia are likely related to the hyperintensity observed in the tectum, mesencephalic tegmentum, cerebellum, and pons. Patients who develop Korsakoff psychosis have extensive diencephalic lesions. Sensitivity and specificity rates for brain MRI for diagnosis of WE are 53% and 93%, respectively [24]. Such high signals typically disappear after therapy with thiamine, as shown by the case herein reported. 

The gold-standard treatment is thiamine replacement, which leads to the resolution of symptoms after a period ranging from a few hours to some weeks, depending on the severity of the disease. If left untreated, WE may progress to develop into WKS, which in turn can bring about symptoms such as amnesia, which take more time to resolve [25,26]. Any patient with hyperemesis gravidarum should receive thiamine supplements. Thiamine doses recommended for parenteral therapy range from 50 to 1200 mg a day for two weeks, generally followed by 100 mg a day orally till delivery. As for the thiamine replacement sufficient to definitely suggest the correct dose, the route of administration, and the duration, not enough evidence from controlled trials is currently available. The European Federation of Neurological Societies recommends a dose of 200 mg i.v. three times per day [27], and such a recommendation is ranked at level C, indicating limited evidence.

In order to stop the progression toward irreversible damage, thiamine should be administered as soon as possible. Some studies on hyperemesis gravidarum reported that treatment reduced the size and intensity of lesions after 6 to 18 weeks and that MRI scans appeared normal about 4 weeks after delivery. Sometimes, magnesium sulfate is recommended in association with thiamine, as magnesium is a co-factor for transketolase. On the contrary, glucose infusion can stave off thiamine deficiency by triggering an increase in thiamine demand. 

When diagnosed and treated quickly, WE has a favorable prognosis. With thiamine replacement, ocular paralysis may improve, usually within one week, after 2–3 mg of thiamine are administered; for such an improvement to be maintained, at least 50 mg should be administered. Nystagmus may continue in 35% of patients. Confusion usually disappears within a month. In about 80% of patients, memory loss may continue for a long time, and in about 35% of patients, gait disorders persist. Mild ataxia and dysmetria may persist [22]. Indeed, ataxia persisted in our patient, too. 

We conducted a review of available research findings as of February 2023 about WE in women with HG. We found a single systematic review by Oudman et al. [23] that included 146 case studies (case reports and case series) reporting on 177 cases from 1955 until May 2018. The authors of said review article evaluated patient characteristics (the mean age, the mean duration of excessive vomiting at diagnosis, and the mean gestational age before onset of WE), the incidence rates of WE prodromal symptoms and classic triad, the percentage of radiologic findings, the rate of suboptimal treatment with thiamine, and fetal and maternal mortality. Moreover, 20 studies centered around 25 cases from June 2018 to February 2023 [28,29,30,31,32,33,34,35,36,37,38,39,40,41,42,43,44,45,46,47] were also accounted for, and we evaluated the overall data of the Oudman review [23] and further subsequent studies for a total of 202 cases. The weighted average age of the patients was 27. The mean duration of excessive vomiting due to HG before the onset of WE symptoms was 6 weeks, while the mean gestational age at diagnosis was 15 weeks. Oudman et al. [23] also point out that patients presenting WE along with mental status alterations tended to be older on average than patients in whom such a change was not observed. The prodromal symptoms of WE (nausea, loss of appetite, and vomiting) were present in all HG cases. The full triad was present in 57.9%. An altered mental status involving confusion, delirium, and problems in alertness or cognition was present in 80.1%. These findings confirm that the cerebellum plays a role not only in motor coordination but also in cognitive capabilities, as demonstrated by some studies [48,49,50]. Ataxia was reported in 76.7% and ocular signs in 81.6%, the most frequent of which was nystagmus (75.2%). A total of 70% of women underwent imaging, the majority at MRI (83%); radiologic findings were present in 69.3%. Only 60.9% of studies reported a plan for thiamine therapy. Pregnancies resulted in spontaneous abortion in 37% of cases, and there were nine cases of maternal deaths (4.5%). Hardly any authors reported data on long-term sequelae, unlike our case. After poring over all available research findings, it became apparent that there still has not been a significant improvement in the management of thiamine deficiency due to hyperemesis gravidarum. In all the cases reported, in fact, the diagnosis of Wernicke encephalopathy was mostly delayed due to underestimation of hyperemesis consequences. In addition to this, it is likely that both the lack of definitive evidence-based guidelines and, in some cases, the lack of adequate control of compliance to long-term therapy, play a crucial role in determining inappropriate surveillance on short-term effects and on long-term sequelae.

### 4.1. The Need for Targeted Guidelines

Overall, from a medicolegal perspective, it is worth bearing in mind that the sound management of WE has distinctively complex traits and features that need a team approach. WE can, in fact, manifest itself in various forms; hence, the patient should be managed by a neurologist and an intensivist, in addition to other specialists, depending on possible organ involvement. Of great relevance are certainly the roles of nurses and dietitians, in addition to pharmacists (many WE patients are managed as outpatients). Since WE patients often suffer from malnutrition, dietary counseling should be undertaken in order to gauge the calorie needs of each patient in a tailored and targeted fashion and provide food intake as well as thiamine supplementation. Alcohol consumption ought to be strongly discouraged, and since therapeutic pathways rely on thiamine, adherence to supplementation plans is of utmost importance, as is the treatment of electrolyte deficiencies. The family setting plays a pivotal role as well: the patient’s relatives should be fully aware of the prognosis and prepare for long-term care if Korsakoff syndrome arises. Close communication between members of the team is vital to ensure that the patient is receiving the current standard of care treatment. The ultimate goal is to improve the quality of life and lessen the burden on the family [46]. Accounting for patients with conditions and/or comorbidities that can expose them to a higher WE risk is also essential. For instance, in addition to conditions such as hyperemesis gravidarum [50,51], bariatric surgery is known to entail a higher risk of nutritional deficiencies. Most available data on WE-related litigation, in fact, involve patients who had undergone bariatric surgery, after which thiamine status follow-up is recommended for at least 6 months. [52,53,54]. The procedure, in fact, revolves around the removal of some parts of the stomach, with consequent possible insufficient thiamine absorption [55,56], which may result in deficiencies and WE, in addition to nutrient/vitamin deficiencies, especially B12 and B1, folate, zinc, and copper [57,58]. Overall, negligence-based malpractice charges can give rise to extremely high compensatory damages involving healthcare professionals and facilities. 

### 4.2. A Reasonable Degree of Objectivity for Medicolegal Tenability

It should therefore be stressed that adherence to evidence-based guidelines and best practices needs to be provable and documented. In this regard, for the sake of thoroughness, it is worth mentioning that WE is diagnosed with a degree of sensitivity that relies on optimization through the already mentioned Caine criteria [12] and that such a degree of sensitivity is close to 100% in patients with alcoholism without hepatic encephalopathy. Hence, a therapeutic pathway, viable from a clinical as well as medicolegal perspective, ought to consider a WE diagnosis in the presence of two of the following features: nutritional deficiency, altered mental state or memory, oculomotor abnormalities, and cerebellar dysfunction [59].

Available guidelines such as those issued by the European Federation of Neurologic Societies (EFNS) [26] provide valuable guidance in the form of recommendations, with levels ranging from B (indicating moderate evidence based on at least one high-quality study or multiple moderate-quality studies) to C (i.e., limited evidence based on at least one study of moderate quality). The most relevant such recommendations have been summarized in Table 1.

The ability to documentably prove adherence to evidence-based recommendations and guidelines may be key to shielding professionals and facilities from negligence-based malpractice allegations, should unfavorable outcomes ultimately occur. 

Particularly under tort-law statutes in many jurisdictions, the onus is, in fact, on professionals/facilities to prove compliance with scientifically established standards of care in order to ensure medicolegal soundness and provide a degree of objectivity and clarity in all prognostic and therapeutic pathways [60]. Such fundamental elements are even more essential in WE management, given the high level of variability in thiamine dosages and therapeutic approaches implemented in WE treatment protocols, despite currently available validated guidelines [27]. As a matter of fact, despite the data highlighting the therapeutic and prognostic value of timely thiamine-based approaches following early diagnosis, a lack of clarity and conclusiveness still lingers as to the possible impact of substantially high thiamine administration on therapeutic supplementation purposes. Treatment standards are unlikely to achieve an acceptably high degree of decisiveness in the short term, and the absence of A-level recommendations reflects such a state of affairs, mostly due to limited currently available data and methodological shortcomings. This makes cautionary approaches and strict compliance with evidence-based standards even more essential from the clinical as well as medicolegal standpoint [61]. 

## 5. Conclusions

Hyperemesis gravidarum is a rare cause of Wernicke encephalopathy, a severe and often misdiagnosed condition. Our report emphasizes how important it is to take into account this complication in clinical practice, with evidence-based guidelines and recommendations to provide guidance [60]. 

The proper management of vomiting in pregnancy, along with the timely replenishment of thiamine stores, can be helpful in treating neurological symptoms and preventing both maternal and fetal morbidity, which can lead to improved pregnancy outcomes. Unfortunately, maternal sequelae may still occur in some cases, which is why long-term follow-up is recommended. A broad-ranging research effort is needed to frame more comprehensive evidence-based guidelines on WE management. Relying on clean-cut, widely-shared standards is, in fact, essential in terms of improving outcomes and providing as high a degree of objectivity as possible for clinical as well as medicolegal soundness. 

## Figures and Tables

**Table 1:** Evidence-based recommendations on WE diagnostics/therapeutics.

Recommendation	Level
Although prevalence is higher in alcoholics, WE should be suspected in all clinical conditions possibly linked to thiamine deficiency. Accounting for the various presentations of clinical signs between alcoholics and nonalcoholics is essential from a diagnostic perspective.	C
WE diagnosis of WE in alcoholic patients must lie on two of the following features: (i) nutritional deficiencies (ii) eye signs, (iii) cerebellar dysfunction, and (iv) either an altered mental state or mild memory impairment.	B
Acute WE diagnosis in both alcoholic and nonalcoholic subjects ought to be confirmed by MRI.	B
Thiamine for the treatment of suspected or manifest WE should be administered preferably intravenously, before any carbohydrate consumption, 200 mg thrice daily.	C
After bariatric surgery, thiamine status follow-up and parenteral thiamine supplementation (also indicated for all patients deemed at-risk and admitted to the ER) should be carried out for at least 6 months.	B
Patients who possibly died from WE should be autopsied.	N.a.

## Data Availability

All data are available upon request to the corresponding author.
